# Granulomatosis With Polyangiitis (GPA): A Case Report

**DOI:** 10.7759/cureus.86710

**Published:** 2025-06-25

**Authors:** Rana Hoseen, Sara G Haroutunian, Salvatore Grasso, Donya Farmand, Marinelle Camilon, Sanaz Hashemi

**Affiliations:** 1 Internal Medicine, Ross University School of Medicine, Miramar, USA; 2 Internal Medicine, California Hospital Medical Center, Los Angeles, USA; 3 Medicine, Ross University School of Medicine, Miramar, USA; 4 Medical Genetics, National Institutes of Health, Bethesda, USA; 5 Family Medicine, California Hospital Medical Center, Los Angeles, USA

**Keywords:** antineutrophil cytoplasmic antibody (anca), conjuctivitis, granulomatosis with polyangiitis (gpa), small infiltrates in the lung, small vessel vasculitis

## Abstract

Granulomatosis with polyangiitis (GPA) is a rare necrotizing vasculitis disease affecting small to medium vessels. The exact cause of GPA is not fully understood, but the inflammation is associated with anti-neutrophil-cytoplasmic-antibody (ANCA). GPA involves the upper and lower respiratory tract, systemic vasculitis, and glomerulonephritis. This case report describes a young female patient presenting with a dry cough for a month that did not improve with Bactrim, weight loss, sinusitis, mastoid effusion, and multiple mass-like infiltrates in the lungs on imaging, who was found to have GPA vasculitis after being confirmed with a biopsy.

## Introduction

Vasculitis is an autoimmune disorder in which the body's defense system attacks healthy tissues, such as granulomatosis with polyangiitis (GPA) vasculitis [1]. GPA is a small to medium vessel inflammation associated with anti-neutrophilic-cytoplasmic-antibody (ANCA). ANCA activates neutrophils, which increases their adherence to the endothelium to form neutrophilic microabscesses that lead to the formation of granulomas. These granulomas will cause partial or total occlusion of blood vessels, decreasing the blood flow to distal organs [2]. Researchers believe an infection might contribute to the onset of GPA in addition to environmental and genetic factors [1]. GPA affects the blood vessels of the nose, sinuses, throat, lungs, and kidneys [3]. The first case reported was by a German medical student in 1931.

The severity of the symptoms of GPA varies from one person to another; some patients might have mild symptoms, while others might develop severe or life-threatening symptoms [1]. Patients usually present with nonspecific symptoms such as fever, weight loss, and myalgia. Also, 90% of the patients will have upper respiratory tract problems such as sinusitis, crusting rhinitis, otitis media, mastoiditis, and hearing loss. Nearly 50% of patients present with bilateral or unilateral pulmonary infiltrates. About 50-60% of the patients have dermatological involvement, including purpura of the lower extremities. About 10-20% of patients have rapidly progressive glomerulonephritis leading to chronic kidney disease or end-stage renal disease. Finally, half of the patients have eye involvement, such as developing conjunctivitis or scleritis [2].

Diagnosis requires blood and urine tests, imaging studies, and a biopsy to confirm the diagnosis. Blood tests will show elevated C-reactive protein (CRP), erythrocyte sedimentation rate (ESR), and positive ANCA. Urine tests will show abnormal results, including positive red blood cells (RBC) or elevated protein, to indicate affected kidneys [2]. Computed tomography (CT) and chest X-rays (CXR) will show lesions or hemorrhages in the lungs [2]. Lastly, lung or renal biopsy is the definitive method of confirming GPA.

## Case presentation

A 27-year-old female with no significant past medical history presented to the emergency department with a dry cough for a month, along with night sweats and weight loss, and failed an oral trimethoprim-sulfamethoxazole course.

**Figure 1:**
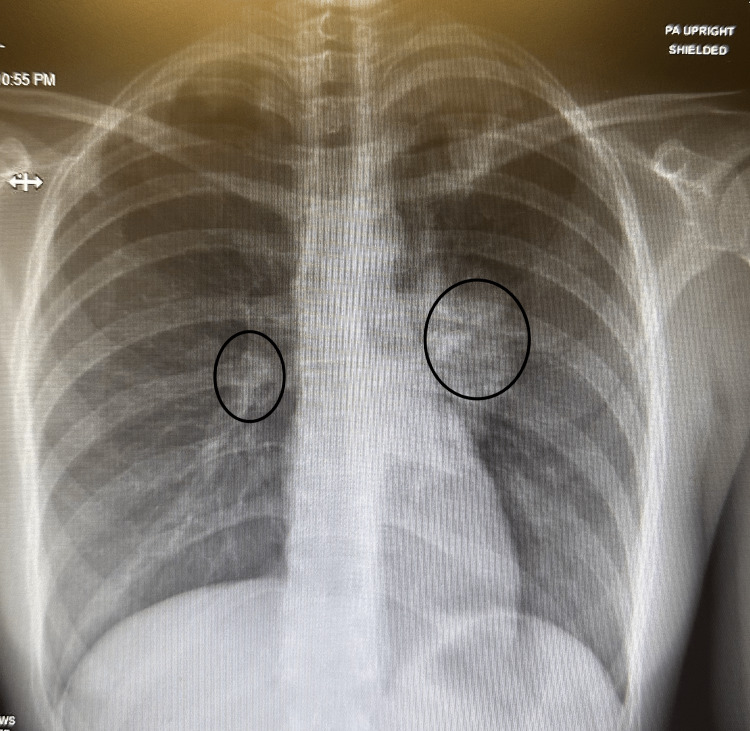
Chest X-rays The circles show mass-like infiltration in the lung parenchyma

**Figure 2:**
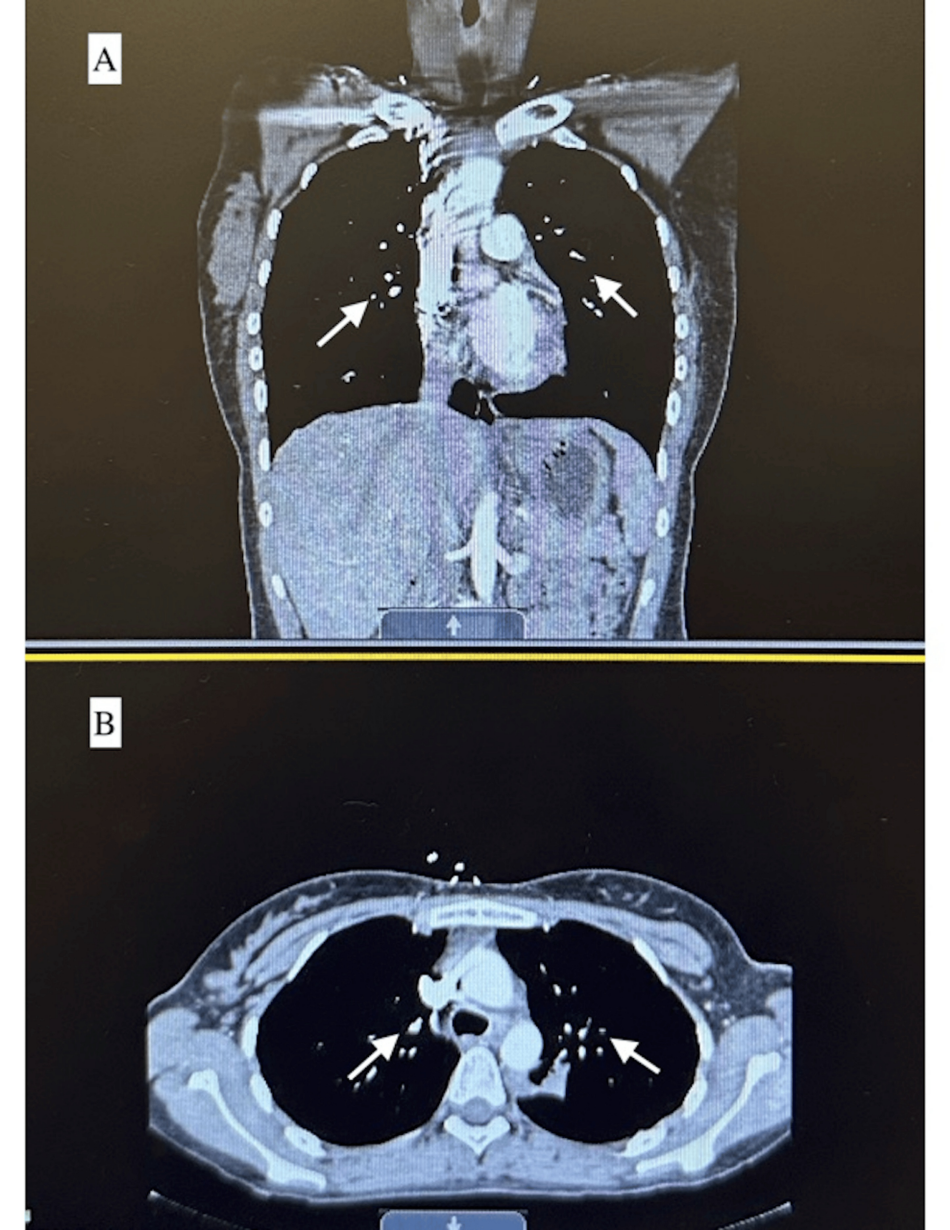
CT of the chest A) Coronal CT scan of the chest that shows mass-like infiltrates within the left and right lung cavities (white arrows); B) Axial CT scan of the chest that shows mass-like infiltrates within the left and right lung cavities (white arrows)

On admission, the patient had leukocytosis (23.3 cells/μL) and tachycardia (130 bpm), as seen in Table 1 below. CXR and CT angiography of the chest showed multiple mass-like lung infiltrates as seen in Figures 1 and 2. The patient was admitted for further evaluation. Infectious disease (ID) was consulted, and the patient was started on empiric intravenous antibiotics (IV Abx) while cultures were pending, with no improvement. IV Abx was discontinued. A pulmonary consult was recommended for empiric steroids and status post bronchoscopy, and a lung biopsy was suggested if there was no improvement. Cultures from bronchoscopy showed no growth.

**Figure 3:**
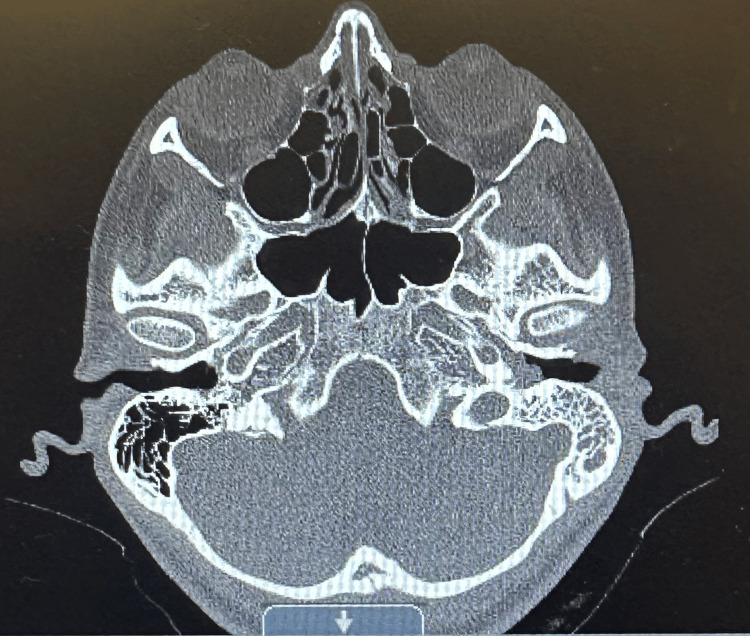
CT of the orbit/stella/posterior fossa without contrast

The patient complained of chronic left mastoiditis, which was seen in CT of the temporal bone, and she was started on Loratadine to help with the symptoms per the ear nose throat (ENT) specialist's recommendation. CT of the orbit showed sinusitis and mastoid effusion but no lymphadenopathy to suggest lymphoma or other malignancy, as shown in Figure 3. ENT recommended an outpatient audiometric evaluation and added fluticasone nasal spray and loratadine for nasal congestion. The patient also had left shoulder pain, but the X-ray was normal. The patient developed conjunctivitis on day four of admission and was treated with neomycin + polymyxin B + dexamethasone ophthalmic drops for five days per ID.

The patient's C-ANCA against PR3 was markedly elevated (492 U/mL), suggesting GPA vasculitis with primary pulmonary and upper airway involvement. On the physical exam, there was no obvious rash on her extremities available for biopsy, and she had no current renal involvement. Therefore, the patient underwent successful video-assisted thoracic surgery (VATS) with wedge resection biopsy on day 12 of admission by cardiothoracic surgery. The pathology of the biopsy confirmed GPA vasculitis, as it showed acute capillaritis and poorly formed epithelioid granulomas. Therefore, the patient began taking steroids and methotrexate. Three days later, a repeat CXR showed a trace left-sided pneumothorax and ill-defined left retrocardiac opacities, suggesting atelectasis.

Nephrology was consulted to evaluate the renal involvement of GPA. The patient had stable renal function throughout admission; however, hematuria was noted on urine analysis, as seen in Table 1 below. The patient was recommended to follow up with outpatient nephrology for further evaluation. The ophthalmology consultant noted that the patient did not have manifestations of vasculitis on the exam, and the redness in the eyes was due to dry eyes. The patient can use eye lubricant to help with symptoms.

**Table 1:** Lab values

Lab values and vital signs	Patient's values	Normal range
Complete blood count (CBC)	Leukocyte of 23.3 thousand/uL	5.0-11.0 thousand/uL
Heart rate (HR)	130 beats per minute (BPM)	60-100 BMP
Urine red blood cells (RBC)	51-100	None

On the day of discharge, the patient was hemodynamically stable, tolerating diet, and ambulating. The patient was advised to follow up with her primary care provider within one week to obtain appropriate outpatient follow-ups with specialists. The patient was discharged with a three-week 30 mg steroid dose, then tapered by 10 mg weekly, and trimethoprim-sulfamethoxazole as prophylaxis, and was given methotrexate and steroids.

## Discussion

GPA is a rare disease that causes inflammation in the blood vessels (vasculitis) and tissues such as the lungs, sinuses, and kidneys, which can lead to organ system damage. It can affect people of all ages, but the peak age group is the 5th-7th decade of life [4]. As a result, physicians may be less likely to consider GPA in younger adults, potentially leading to delayed or missed diagnosis despite the presence of suggestive symptoms. Our patient came in complaining of a month-long history of cough, weight loss, and night sweats, along with elevated ESR and CRP that are suggestive of lung carcinoma, lymphoma, tuberculosis, or systemic vasculitis. Further investigations showed that the patient has positive c-ANCA against PR3, which is highly supportive of vasculitis. Therefore, a biopsy was done and the diagnosis was confirmed.

This patient exhibited a typical presentation of GPA, including sinusitis, a mass-like infiltrate in the lungs on chest CT, cough, nasal congestion, and conjunctivitis. These features align with the most frequently reported symptoms in GPA, as illustrated in Table 1. Although there was no evidence of renal involvement at the time of evaluation, the patient was referred to outpatient nephrology for ongoing monitoring to help prevent the development of progressive glomerulonephritis.

The patient was started on a 30 mg dose of steroids for three weeks, then tapered down by 10 mg weekly due to the patient's low body weight, as well as Methotrexate. Her symptoms were improving with the treatment, and she was advised to follow up with the specialists to manage her disease and prevent any worsening prognosis.

**Table 2:** Comparison between different GPA case reports GPA - granulomatosis with polyangiitis; ESR - erythrocyte sedimentation rate; CRP - C-reactive protein; ANCA - anti-neutrophilic-cytoplasmic-antibody

Comparison Criteria	27 y.o. female (our patient)	62 y.o. female [5]	70 y.o. male [6]	52 y.o. male [7]	66 y.o. male [8]
Generalized symptoms	Cough, weight loss, shoulder pain	Cough, weight loss	weight loss, fatigue, dry cough	Fever, cough, arthralgia	Fever, cough
Upper respiratory symptoms	Mastoiditis, sinusitis	Sinusitis, hearing loss	Otitis media	Runny nose	None
Lower respiratory symptoms	Mass-like infiltrates in the lungs	left lower lobe lung mass	Multiple small infiltrates in both lungs	Reticulonodular shadows scattered all over the lung field	bilateral parenchymal and pleural-based nodules
Kidney	Mild hematuria was noted on UA but had stable renal function	None	None	Proteinuria, pauci-immune deposition of antibodies, mostly IgG, in a linear pattern with crescent formation	Hematuria and proteinuria, segmental necrotizing, and crescentic glomerulonephritis with granulomatous features
Blood vessel	No purpura was seen	None	None	None	None
Lab results	Leukocytosis, elevated ESR and CRP, Positive c-ANCA against PR3	Leukocytosis, elevated ESR, Positive c-ANCA	Leukocytosis, elevated CRP, positive p-ANCA	Elevated ESR, positive p-ANCA	Positive c-ANCA
Treatment	Steroids and methotrexate	Steroids and methotrexate	Steroids and cyclophosphamide	Steroids and azathioprine	Steroids and plasmapheresis, then rituximab was added

## Conclusions

This case highlights an uncommon presentation of granulomatosis with polyangiitis (GPA) in a young woman, characterized by prominent respiratory involvement without renal dysfunction or vasculitic symptoms. GPA, though more prevalent in older adults, should be considered in patients of all ages who present with unexplained respiratory symptoms, systemic features, and radiologic lung findings. Early diagnosis, ideally confirmed through tissue biopsy, is critical to initiate timely immunosuppressive therapy and prevent disease progression. Treatment typically involves corticosteroids combined with immunosuppressive agents such as methotrexate or cyclophosphamide, followed by maintenance therapy to minimize relapse risk. This case underscores the importance of maintaining a broad differential diagnosis and close monitoring, even in atypical patient populations.
